# Prediction of Infarct Growth and Neurological Deterioration in Patients with Vertebrobasilar Artery Occlusions

**DOI:** 10.3390/jcm9113759

**Published:** 2020-11-22

**Authors:** Seungyon Koh, Ji Hyun Park, Bumhee Park, Mun Hee Choi, Sung Eun Lee, Jin Soo Lee, Ji Man Hong, Seong-Joon Lee

**Affiliations:** 1 Department of Neurology, Ajou University School of Medicine, 164, World cup-ro, Yeongtong-gu, Suwon, Gyeonggi-do 16499, Korea; esin4498@gmail.com (S.K.); choimoonhee09@gmail.com (M.H.C.); plumpboy@hanmail.net (S.E.L.); jinsoo22@gmail.com (J.S.L.); dacda@hanmail.net (J.M.H.); 2 Office of Biostatistics, Medical Research Collaborating Center, Ajou Research Institute for Innovative Medicine, Ajou University Medical Center, Suwon, Gyeonggi-do 16499, Korea; jhn1105@gmail.com (J.H.P.); bhpark@ajou.ac.kr (B.P.); 3 Department of Biomedical Informatics, Ajou University School of Medicine, Suwon 16499, Korea

**Keywords:** basilar artery, brain ischemia, intracranial atherosclerosis, embolism, infarction

## Abstract

We aimed to identify predictors of infarct growth and neurological deterioration (ND) in vertebrobasilar occlusions (VBOs) with a focus on clinical-core mismatch. From 2010 to 2018, VBO patients were selected from a university hospital registry. In total, 138 VBO patients were included. In these patients, a posterior circulation Alberta Stroke Program Early CT score (PC-ASPECTS) less than 6 was associated with futile outcome. Within patients with feasible cores, a decrease in PC-ASPECTS score of 2 or more on follow-up imaging was classified as infarct growth and could be predicted by a National Institutes of Health Stroke Scale (NIHSS) mental status subset of 1 or higher (odds ratio (OR): 3.34, 95% confidence interval (CI) (1.19–9.38), *p* = 0.022). Among the 73 patients who did not undergo reperfusion therapy, 13 patients experienced ND (increase in discharge NIHSS score of 4 or more compared to the initial presentation). Incomplete occlusion (vs. complete occlusion, OR 6.17, 95% CI (1.11–34.25), *p* = 0.037), poorer collateral status (BATMAN score, OR: 1.91, 95% CI (1.17–3.48), *p* = 0.009), and larger infarct cores (PC-ASPECTS, OR: 1.96, 95% CI (1.11–3.48), *p* = 0.021) were predictive of ND. In patients with VBO, an initial PC-ASPECTS of 6 or more, but with a decrease in the mental status subset of 1 or more can predict infarct growth, and may be used as a criterion for clinical-core mismatch. ND in VBO patients presenting with milder symptoms can be predicted by incomplete occlusion, poor collaterals, and larger infarct cores.

## 1. Introduction

In anterior circulation stroke, endovascular treatment (EVT) for emergent large vessel occlusion is a well-established treatment. The indication is relatively clear, and its effectiveness has been proven through numerous randomized controlled trials (RCTs) [1]. While there have been no successful RCTs demonstrating the effectiveness of EVT in vertebrobasilar occlusion (VBO), it is strongly recommended that EVT be used for treating VBO [2]. In practice, however, decisions for EVT in VBO patients are complicated by diversity in clinical courses, such as prodromal symptoms, late progressions, and a wide range of clinical symptoms despite similar-looking vascular occlusions. Thus, there are some issues to be further resolved for EVT of VBO.

One major issue is generation of a clinical-core mismatch criterion in VBO. Time-based selection criteria for EVT in VBO may be complicated by heterogeneity in clinical presentation [3], and a clinical-core mismatch criterion can be supportive. The clinical-core mismatch criterion is used to identify patients with significant neurologic deficits but with limited infarct core that may benefit from EVT regardless of stroke onset-to-door time [4]. The clinical-core mismatch can predict infarct growth and maximize the treatment effect of EVT [5]. For example, the original reports have described an inclusion criteria of National Institutes of Health Stroke Scale (NIHSS) ≥10 with an infarct core of ˂31cc for anterior circulation large vessel occlusion patients [4]. In the anterior circulation, clinical-core mismatch is widely accepted as a predictor of infarct growth along with other methods such as diffusion-perfusion mismatch [6], or collateral status [7]. Theoretically, a clinical-core mismatch criteria can be superior to diffusion-perfusion mismatch or collateral status in VBO. Perfusion imaging of the posterior circulation is limited by its low spatial resolution, while leptomeningeal collateral assessment via noninvasive methods cannot be applied to the posterior circulation. However, whether to use the total NIHSS score, or to use special subsets of neurological deficits for the mismatch criteria to predict infarct growth, can be debatable. In the anterior circulation, cortical signs such as aphasia, extinction, and gaze deviation may be considered as a marker of hemispheric hypoperfusion [5]. It is not clear which neurological signs may represent this role in the posterior circulation.

A second major issue is neurological deterioration (ND). For VBO, even for patients that present with lower clinical severity, there is a high rate of neurological deterioration (ND). High rates of intracranial atherosclerotic occlusions are partly responsible. Owing to its longstanding diseased vessel status, patients with intracranial atherosclerotic occlusions may present with a wide variety of clinical severity, and experience a high rate of ND. While the pathomechanism underlying ND is diverse, late perfusion failure following ND are comparatively more common and often lead to devastating neurological outcomes. However, in VBO the actual frequency, the distributions of pathomechanism of the ND, and potential predictors are not well reported. Identification of such factors are needed to evaluate the feasibility of delayed or even preventive EVT.

The fact that there are high rates of intracranial atherosclerotic occlusions [7] in VBO is associated with both issues. However, such associations have not been appropriately addressed in previous studies; past literature describing clinical behaviors of VBO with regard to pathophysiology date back to the pre-EVT era [8], while reports of EVT in VBO seldom address the heterogeneity in clinical presentation. Further, the underlying occlusion etiology is difficult to verify in cases that do not undergo EVT, for identification of underlying stenosis can only be performed after recanalization is achieved [9]. However, recent studies focusing on pre-EVT identification of intracranial atherosclerotic occlusions have reported occlusion type analysis based on the idea that an embolus would likely become lodged at the site of an arterial bifurcation rather than being halted in the middle of the artery [10]. This etiologic classification was shown to be well-matched with post-procedural diagnosis especially in the VBO population [11], and used as a surrogate for intracranial atherosclerotic occlusions in both the anterior [12] and posterior [13] circulations.

Thus, in VBO patients, we aimed to describe the overall clinical picture that may benefit from reperfusion therapies, by addressing the issue of infarct growth and neurological deterioration. To achieve this goal, this study identified VBO patients based on presenting angiographic imaging and tissue imaging, rather than therapeutic modality or presenting time. In this population, we first aimed to generate a clinical-core mismatch criteria through identification of futile infarct cores and predictors of infarct growth, correcting for occlusion type. The total NIHSS score and its subsets were compared in this analysis. Second, in patients that did not undergo revascularization, the frequency, pathomechanism of ND, and its predictors were evaluated.

## 2. Materials and Methods

The data that support the findings of this study are available from the corresponding author, upon reasonable request.

### 2.1. Patient Selection

**Figure 1:**
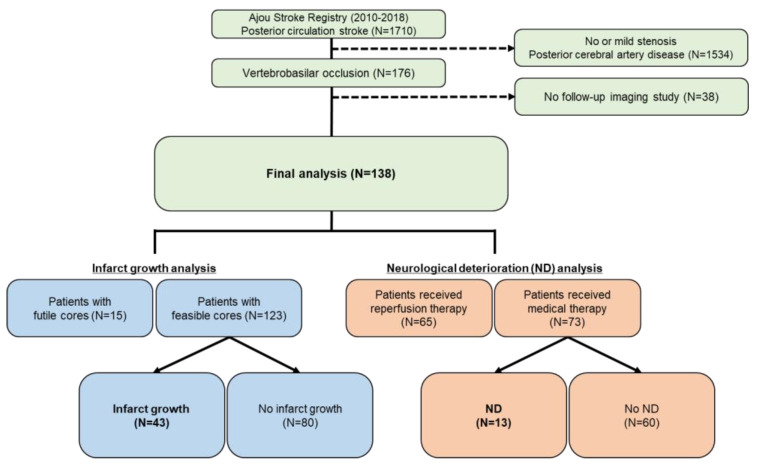
Flow chart of the patient selection process is provided in this flowchart. ND, neurological deterioration.

The flow chart for patient inclusion is summarized in Figure 1. From January 2010 to December 2018, all posterior circulation stroke patients were identified from Ajou Stroke Registry, a prospectively collected stroke registry from a university hospital stroke center. All patients admitted to the department of neurology for the treatment of acute ischemic stroke were registered regardless of the treatment or imaging. The basic demographics, the initial and follow-up NIHSS score at 2 h and 1, 3, 7, and 14 days, and the day of discharge, functional outcome as measured by modified Rankin scale (mRS) at discharge and at 3 months, laboratory tests, and the location of the infarction were collected from the registry. In all posterior circulation stroke patients included in the registry, patients with VBO was identified through medical record search for keywords “occlusion”, or “stenosis” in baseline CTA or MRA reports, and further reviewed by two investigators (S.K., senior resident and S.-J.L., interventional neurologist) to identify an occlusion or near total occlusion in the basilar artery, bilateral vertebral artery, or dominant vertebral artery with no contralateral vertebral artery flow. Near total occlusions in which antegrade blood flow cannot be ascertained, or only minimal flow is suspected, was included because similar looking lesions present with a wide range of clinical severity, and in a large number of cases, antegrade blood flow cannot be ascertained without conventional angiography. Patients who had data regarding the initial and final infarct volumes available, which was assessed magnetic resonance imaging (MRI) or CT, were selected. Ethics approval was obtained from the local institutional review board, and the board waived the need for patient consent.

### 2.2. Variables and Image Analysis

**Figure 2:**
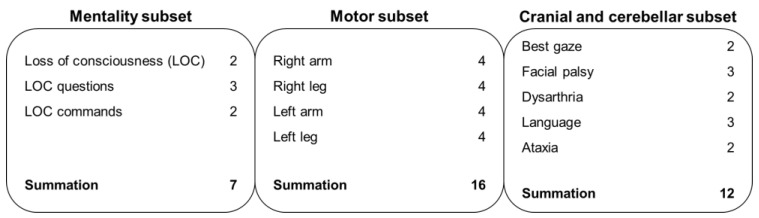
Classification of the NIHSS subsets for mental status, motor, and cranial and cerebellar scores.

Variables being investigated were obtained retrospectively by reviewing medical records. The NIHSS was divided into mental status, motor, and cranial and cerebellar subsets. The mental status subset included level of consciousness (LOC), LOC questions, and LOC commands; the motor subset included bilateral arm and leg scores; and the cranial and cerebellar subset included best gaze, facial palsy, dysarthria, language, and ataxia scores. This subset classification is summarized in Figure 2. Prodrome was defined as a preceding fluctuation of neurological symptoms suggestive of a vertebrobasilar transient ischemic attack.

**Figure 3:**
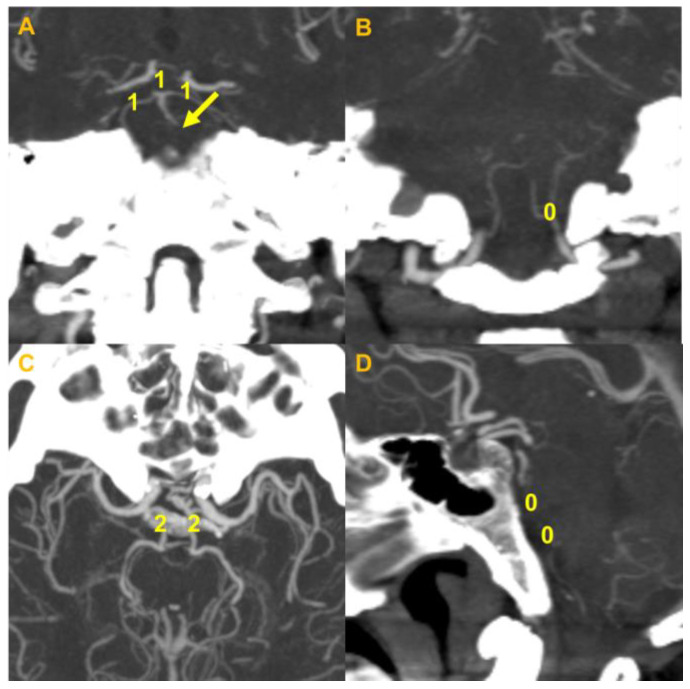
An example of a BATMAN score from a truncal-type occlusion from the cohort. (**A**) Coronal reconstruction of the MIP image of the basilar artery. Occlusion at the vertebrobasilar artery with visualization of the distal part and the basilar top is seen. Both PCAs are visualized. For the BATMAN score, each 1 point is given for the distal BA, and both PCAs. (**B**) Coronal MIP image of bilateral VAs. Note that both VAs are occluded. Zero point was given. (**C**) Axial MIP image showing both posterior communicating arteries. Two points for each communicating artery was given. (**D**) Sagittal MIP image showing occluded proximal and mid-BA. Zero points were given for each part of the BA, constituting a total BATMAN score of 7. BATMAN, Basilar Artery on Computed Tomography Angiography; BA, basilar artery; MIP, maximal intensity projection; PCA, posterior cerebral artery; VA, vertebral artery.

**Figure 4:**
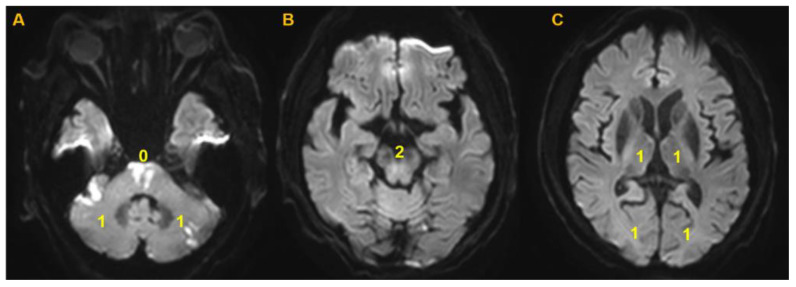
An example of a calculation of PC-ASPECTS from diffusion-weighted image. (**A**) Diffusion restriction is seen at the pontine level. Two points were subtracted at this level. Even though scattered infarction was noted in the bilateral cerebellum, lesions did not exceed 20% of the territories. (**B**) No acute infarction was noted at the midbrain level. (**C**) No acute infarction was noted in the bilateral thalami and PCA territories. This constitutes a total score of 8. PC-ASPECTS, Posterior Circulation-Alberta Stroke Program Early Computed Tomography Score; PCA, posterior cerebral artery.

The image analyses were performed using commercial image-viewing software (Picture Archiving and Communication System; Maroview 5.3 Infinitt Co., Seoul, Korea). It was performed by two investigators (S.K. and S.-J.L.) who were blinded of clinical information at the time of analyses. Disagreement was resolved by consensus. The location of the occlusion and the degree of occlusion were analyzed. For baseline infarct volume measurements, diffusion-weighted imaging (DWI) on MRI was used for all patients. For follow-up infarct volumes, DWI was utilized primarily, and non-contrast CT was used when follow-up MRI was not available. Follow-up imaging was performed within a week after the initial study. The infarct volume was semiquantitatively graded using a previously described scoring system: posterior circulation Alberta Stroke Program Early CT score (PC-ASPECTS) [14]. This scoring system is the posterior circulation-equivalent of Alberta Stroke Program Early CT Score used in the anterior circulation stroke, composed of 8 territories that are supplied by the vertebrobasilar vasculature. In comparison to the anterior circulation, higher points are given to the brainstem, and due to bone artifact or partial volume effect, it is commonly measured in MR DWI, where it has proven its predictive power [15]. PC-ASPECTS was scored as previously described, subtracting each assigned points when more than 20% involvement was found in the relevant territory [16]. Infarct growth was defined as a decrease in PC-ASPECTS of ≥2 points from the initial imaging study to the follow-up imaging study. Occlusion degree was classified into incomplete occlusion and complete occlusion based on CTA maximal intensity projection images or magnetic resonance angiography (MRA); it was classified as a complete occlusion when anterograde luminal flow was definitely missing and as an incomplete occlusion when the presence of anterograde luminal flow was uncertain or minimal flow was suspected. Occlusion types were classified into truncal type occlusion (TTO) suggestive of intracranial atherosclerotic occlusions and branching site occlusion (BSO) suggestive of embolic occlusions [7,17]: Non-visualization of the basilar artery bifurcation site due to thrombus indicates BSO, and sparing of the bifurcation site indicates TTO [11]. Baseline collateral status and thrombus burden were also evaluated based on CTA or MRA using the Basilar Artery on Computed Tomography Angiography (BATMAN) score [18]. An example of determining the PC-ASPECTS and BATMAN score in a patient is presented in Figure 3 and Figure 4.

### 2.3. Identification of the Clinical-Core Mismatch Criteria That Predicts Infarct Growth

For the generation of the clinical-core mismatch, the core criterion and clinical criterion was sequentially generated. For the core criterion, the futile core was calculated with PC-ASPECTS, by generating an initial PC-ASPECTS score that is highly specific to result in futile outcomes (3 months mRS 5-6) irrespective of treatment. Infarct volumes that do not meet the futile core criterion were considered feasible cores, and clinical mismatch criterion was evaluated within these patients. Significant cut-off values of NIHSS scores and subset scores were generated, which was predictive of infarct growth. For this, area under the receiver operating characteristic curve (AUC) analysis was performed along with expert opinion. Among the clinical scores, the best parameter predictive of infarct growth with other potentially significant variables included in multiple logistic regression analysis was identified as an optimal clinical-core mismatch criterion. The occlusion type was included in the logistic regression analysis to account for occlusion etiology.

### 2.4. Identification of Factors Predictive of Neurological Deterioration

In patients that did not perform EVT, the presence of ND was assessed. ND was defined as an increase in the NIHSS score by 4 or more points between the point of admission and discharge [19]. ND was classified according to its pathomechanism. Clinical profile and imaging findings were compared between patients who experienced ND and those who did not. Multiple logistic regression analysis was performed, including clinically important variables, to identify the independent predictors of ND.

### 2.5. Statistical Analysis

Variables are expressed as numbers (percentage), and median (interquartile range) value. Continuous variables were compared using Student’s t-test or the Mann-Whitney U test. Categorized variables were compared using the chi-square test or Fisher’s exact test. Normality of the distribution was assessed using the Kolmogorov-Smirnov test. Multiple logistic regression was performed for identification of best clinical-core mismatch criteria predictive of infarct growth and for identification of predictors of ND. Multiple logistic regression analysis included clinically important variables. For the multivariate analysis, predictive power was calculated for statistically significant variables. We calculated the power at the 0.05 significance level with a two-sided test for a multiple logistic regression model by using formulae given by Hsieh (1998) [20]. For this analysis, a power of 80% is generally considered acceptable in terms of sample size. Data are presented as the mean ± standard deviation, number (%), or median [interquartile range (IQR)] as appropriate for data type and distribution. All statistical analyses were performed using IBM SPSS Statistics version 25 (IBM Corp., Armonk, NY, USA) and R version 3.6.3. A *p*-value < 0.05 was considered statistically significant.

## 3. Results

From January 2010 to December 2018, 1710 posterior circulation stroke patients were identified. Patients that did not fulfill the criteria for CT-based VBO were excluded, leaving 176 VBO patients. All patients underwent MRI at admission. Among them, 138 patients who had follow-up imaging study using MRI (77/138, 55.8%) or CT (61/138, 44.2%), were selected and included in the analysis. Median follow-up imaging interval was 4 (IQR, 2–5) days.

### 3.1. Infarct Growth and Generation of Clinical-Core Mismatch Criteria

#### 3.1.1. Generation of Core Criterion

In the 138 patients, infarct core was analyzed with PC-ASPECTS. Decreases in PC-ASPECTS could significantly predict futile outcomes with an AUC of 0.761 (0.680–0.842), and the dichotomized PC-ASPECTS score of less than 6 showed a sensitivity of 25.4% and specificity of 100% for futile outcomes irrespective of treatment. Fifteen patients presented with a PC-ASPECTS score of less than 6. The rest 123 patients were considered to present with feasible cores for reperfusion therapy, and were included in the clinical criterion analysis.

#### 3.1.2. Generation of Clinical Criterion That Can Predict Infarct Growth

In the 123 patients (mean age: 67 ± 13; male: 85/123, 69.1%), 43 (35.0%) patients met the infarct growth definition. When patients with infarct growth and those without were compared, infarct growth group had lower rates of male sex, (53.5% vs. 77.5%, *p* = 0.006) and higher NIHSS scores at presentation (18 (8–22) vs. 7 (3–16), *p* = 0.007). Patients presenting with a NIHSS score 11 or more were significantly more frequent in the infarct growth group (67.4% vs. 40.0%, *p* = 0.004), as was mental status subset score of 1 or higher (83.7% vs. 51.2%, *p* < 0.001), motor subset score of 5 or higher (58.1% vs. 27.5%, *p* = 0.001), and cranial and cerebellar subset score of 4 or higher (74.4% vs. 48.8%, *p* = 0.006). Location of occlusion of the distal or proximal basilar artery (BA) was more frequently observed in the infarct growth group, in contrast to the higher number of vertebral artery (VA) occlusions in the non-infarct growth population (*p* = 0.014). In patients that experienced infarct growth, EVT was more frequently performed (60.5% vs. 36.3%, *p* = 0.010), representing patient selection in clinical practice. A good outcome was much less frequently observed in the infarct growth group (14.0% vs. 65.0%, *p* < 0.001). The two groups did not differ in terms of occlusion types (for TTO, 55.8% vs. 72.5%, *p* = 0.061), PC-ASPECTS (9 (8–10) vs. 9 (8–10), *p* = 0.928), or BATMAN scores (5 (3–7) vs. 6 (5–8), *p* = 0.090) (Table 1).

When adjusted for age, sex, occlusion type, reperfusion therapy, BATMAN, and PC-ASPECTS, only mental status subset score of 1 or higher significantly predicted infarct growth (OR: 3.34, 95% CI (1.19–9.38), *p* = 0.022, predictive power: 93.65%), while total NIHSS, motor subset, and cranial and cerebellar subset cut-off values did not (Table 2).

### 3.2. Neurological Deterioration and Its Predictors

Among the 138 total population, 73 VBO patients (age: 68 ± 14; male: 54/73, 74.0%) did not receive reperfusion therapy. The patients presented with a median NIHSS score of 5 (2–15), and a median time interval of 7 (3–26) hours. The majority of the population presented with a TTO (54/73, 74.0%) suggestive of intracranial atherosclerosis. Predictors of neurological deterioration were evaluated in them. In the 73 patients, ND occurred in 13 (17.8%) patients. Causes of ND was infarct growth in 8 (61.5%), lacunar progression in 4 (30.8%), and brainstem compression in 1 (7.7%). Table 3 summarizes the differences in clinical and imaging characteristics between the ND group and those without ND. When patients with ND and no-ND were compared, there were no differences in baseline demographics, except for a higher rate of prodrome (46.2% vs. 11.7%, *p* = 0.003). There were no differences in NIHSS total or subset scores or onset-to-door time. Only 2/13 (15.4%) of the patients that experienced ND could achieve good outcomes, compared to 38/60 (63.3%) in the no-ND group (*p* = 0.002).

Incomplete occlusion compared to complete occlusion was more frequently observed in the ND group (46.2% vs. 16.7%, *p* = 0.020). The initial infarct volume represented by PC-ASPECTS and collateral status represented by the BATMAN score did not differ in the univariate analysis. In the multivariate analysis for prediction of ND, an incomplete occlusion (OR: 6.17, 95% CI (1.11–34.25), *p* = 0.037, predictive power: 54.31%), decreases in collaterals as measured by BATMAN scores (per 1 point decrease, OR: 1.91, 95% CI (1.17–3.11), *p* = 0.009, predictive power: 45.43%), and larger infarct cores measured by decreases in PC-ASPECTS (OR: 1.96, 95% CI (1.11–3.48), *p* = 0.021, predictive power: 12.86%) could predict neurological deterioration with age, onset-to-door time, and NIHSS score at presentation as covariables (Table 4).

## 4. Discussion

In this retrospective study, we found that in VBO patients, an initial PC-ASPECTS of 6 or more, but with a decrease in the mental status subset of 1 or more can predict infarct growth, and may be used as a criterion for clinical-core mismatch. In patients who did not receive reperfusion therapy, an incomplete occlusion rather than complete occlusion based on CT angiography, a poorer collateral circulation status, and larger initial infarct cores were predictive of ND.

The current study has some clinical implications. It is to our knowledge, the first study to generate a clinical-core mismatch criterion in VBO. It is highly likely that EVT for VBO will significantly improve patient outcomes even in the late time window [21], and this criteria may be able to guide patient selection. This criteria may also guide patient selection in the early time window, especially when the NIHSS score is lower. In contrast to the anterior circulation, trials even with contemporary EVT for VBO have shown negative results [22,23]; however, in the recent BASICS trial, subgroup analysis showed that there was a significant difference in outcome favoring EVT in patients with moderate to severe stroke, or NIHSS score ≥ 10 [23], which emphasizes the value of patient selection for clinical trial success. An NIHSS score ≥ 11 was used as a potential cut-off point in our study but did not reach statistical significance. In contrast, a decrease in mental status could significantly predict infarct growth. Often, the NIHSS cut off for performing thrombolysis and EVT is arbitrary, even in the anterior circulation. The presence of cortical signs may guide decision in the anterior circulation, for it can be considered a marker of hemispheric hypoperfusion [5]. Our results suggest that decreases in mental status may be considered as such a marker in the posterior circulation, and can be a more intuitive approach for reperfusion decisions.

The causes of ND, and its predictors also suggest potential clinical implications. In patients that did not undergo immediate reperfusion therapies, the majority of patients were TTO. Therefore, intracranial atherosclerotic pathology would have been responsible for ND in a large number of cases. When we take a look at the causes of ND, the major cause of ND was growth in infarct size. This may occur from arterial embolism, in situ thrombosis, or hemodynamic insufficiency [24]. The second most common cause, lacunar progression, would also be due to branch occlusion within the atherosclerotic plaque. ND could be predicted by incomplete occlusions and poor collaterals. Such patients may benefit from delayed EVT if the patient progresses, or can be potential candidates for preventive intra-arterial tirofiban injection [25], for it can stabilize the irritable plaque surface safely [11].

This study failed to show an association between collateral scores and future infarct growth. Collaterals [26] in the anterior circulation is a major parameter that determines the extent and speed of infarct growth. The BATMAN score measures the collateral status along with the thrombus burden [18]. However, the abundance of atherosclerotic VBO in this study may have diluted the significance of collaterals since atherosclerotic occlusions result in higher collaterals [27] while presenting with a wide range of clinical severity. BATMAN scores, however, predicted ND in a more homogenous set of VBO patients, who were largely atherosclerotic and presented with milder symptoms. Furthermore, the Western literatures, which are most likely comprised of a more homogenous population of embolic VBO, continuously reported the prognostic value of collaterals [18] and the use of collaterals as a marker for response to EVT in extended time windows [21]. Such findings show that the interpretation of collaterals should be performed in a homogenous set of occlusion etiologies.

Interestingly, an incomplete occlusion was not predictive of a less likelihood of infarct growth, but was associated with later neurological deterioration. An incomplete occlusion was classified as possible or uncertain minimal antegrade flow based on CT angiography maximal intensity projection images because similar-looking incomplete atherosclerotic VBO lesions may present with a wide range of clinical symptoms. Indeed, differences in complete or incomplete occlusion did not predict infarct growth. It was, however, predictive of ND, suggesting that patients with critically reduced antegrade flow may be hemodynamically and neurologically unstable compared to patients with chronic complete occlusions, in which collaterals have already been developed.

The present study has some limitations. First, the initial patient selection was based solely on imaging criteria without consideration of clinical severity or time metrics. Thus, the VBO group included in this study differs from literatures that include more homogenous population of VBO that received EVT. This selection criteria for VBO was used to generate a clinical criteria predictive of infarct growth. However, this may result in heterogeneity in disease etiology and treatment course, and over-representation of atherosclerotic VBO. Accordingly, the results of this study needs to be interpreted with care. Second, while only MR DWI was used for the initial PC-APSECTS scores, both MRI and CT was used for the follow-up PC-ASPECTS grading. As CT evaluation of the brainstem can be limited by bone artifact or partial volume effect [28], this must be mentioned as a limitation of this study. However, we believe that this factor would not have significantly influenced the results of the study for two reasons. At the time of follow-up imaging, ischemic lesions change to frank hypodensity, which is more easily visualized in the brainstem then early ischemic changes. Further, PC-ASPECTS is evaluated only in the pons and midbrain of the brainstem and does not include the medulla, where the brainstem ischemia is most difficult to visualize by CT. Third, for the analysis of clinical-core mismatch, futile infarct cores should ideally be evaluated in patients with timely successful reperfusion. However, due to the limited number of VBO patients, a futile infarct core was generated using the total population, and a PC-ASPECTS cutoff which was highly specific for futile outcomes were used instead. This is apparently a limitation of the study. Last, due to the small number of patients included, the statistical power of the multivariable analysis in identification of neurological deterioration is lower than generally accepted. In this regard, the values generated in this study need to be validated in further studies.

## 5. Conclusions

Infarct growth in VBO can be predicted by clinical-core mismatch, especially any decreases in mental status in patients with feasible PC-ASPECTS scores. Neurological deterioration is encountered in VBO patients who present with minor symptoms and can be predicted by incomplete occlusion rather than complete occlusion along with poorer collateral status and larger initial infarct core. These results need to be externally validated in larger cohorts. The value of the clinical-core mismatch criteria in maximizing VBO EVT efficacy needs to be validated in a prospective trial.

## Figures and Tables

**Table 1:** Comparison of patients who experienced infarct growth and who did not.

Variables	Infarct Growth (*N* = 43)	No Infarct Growth (*N* = 80)	*p* Value
**Age**	72 (59–80)	66 (55–75)	0.114
**Sex, male**	23 (53.5%)	62 (77.5%)	0.006
**HTN**	21 (48.8%)	47 (58.8%)	0.292
**DM**	13 (30.2%)	22 (27.5%)	0.749
**Atrial fibrillation**	12 (27.9%)	17 (21.3%)	0.407
**Presence of prodrome**	8 (18.6%)	11 (13.8%)	0.477
**Onset-to-door time (h)**	3 (1–7)	3 (2–11.75)	0.857
**NIHSS at presentation**	18 (8–22)	7 (3–16)	0.007
NIHSS ≥11	29 (67.4%)	32 (40.0%)	0.004
**Subset mental status scores**	4 (1–6)	1 (0–4)	0.065
Mental status ≥1	36 (83.7%)	41 (51.2%)	<0.001
**Subset motor scores**	6 (2–8)	2 (0–5.75)	0.003
Motor ≥5	25 (58.1%)	22 (27.5%)	0.001
**Subset cranial and cerebellar scores**	**5 (3–7)**	3 (2–6)	0.048
Cranial and cerebellar ≥4	32 (74.4%)	39 (48.8%)	0.006
**Occlusion degree**			0.518
Complete occlusion	34 (79.1%)	67 (83.8%)	
Incomplete occlusion	9 (20.9%)	13 (16.3%)	
**Occlusion location**			0.014
distal BA	11 (25.6%)	10 (12.5%)	
proximal BA	23 (53.5%)	33 (41.3%)	
VA	9 (20.9%)	37 (46.3%)	
**Occlusion type**			0.061
Truncal-type occlusion	24 (55.8%)	58 (72.5%)	
Branching-site occlusion	19 (44.2%)	22 (27.5%)	
**PC-ASPECTS**	9 (8–10)	9 (8–10)	0.928
**BATMAN**	5 (3–7)	6 (5–8)	0.090
**Reperfusion (EVT ± IV thrombo-lysis)**	26 (60.5%)	29 (36.3%)	0.010
**Final PC-ASPECTS**	5 (3–7)	9 (8–10)	<0.001
**Good outcomes (mRS 0–2)**	6 (14.0%)	52 (65.0%)	<0.001

Numbers are represented by numbers (percentage), median value [interquartile range]; HTN, hypertension; DM, diabetes mellitus; NIHSS, National Institutes of Health Stroke Scale; BA, basilar artery; VA, vertebral artery; PC-ASPECTS, Posterior Circulation-Alberta Stroke Program Early Computed Tomography Score; BATMAN, Basilar Artery on Computed Tomography Angiography score; EVT ± IV thrombolysis, endovascular treatment with or without intravenous thrombolysis; mRS, modified Rankin scale.

**Table 2:** Comparison of logistic regression models for predicting infarct growth using cut-off values generated by NIHSS and its subset scores.

	Variables	OR	95% CI	*p* Value
**Model 1**	NIHSS at presentation≥11	2.16	0.83–5.65	0.116
	Age	1.01	0.98–1.05	0.570
	Sex	2.39	0.96–5.91	0.060
	TTO (vs. BSO)	0.93	0.35–2.43	0.876
	EVT ± IV thrombolysis	1.58	0.66–3.79	0.309
	PC-ASPECTS (per 1 point decrease)	0.93	0.65–1.33	0.682
	BATMAN (per 1 point decrease)	1.16	0.92–1.46	0.220
**Model 2**	Subset mental status ≥ 1	3.34	1.19–9.38	0.022
	Age	1.01	0.98–1.05	0.586
	Sex	2.19	0.88–5.43	0.092
	TTO (vs. BSO)	1.00	0.38–2.65	0.996
	EVT ± IV thrombolysis	1.48	0.61–3.55	0.383
	PC-ASPECTS (per 1 point decrease)	0.95	0.67–1.34	0.760
	BATMAN (per 1 point decrease)	1.17	0.92–1.47	0.200
**Model 3**	Subset motor ≥ 5	2.44	0.96–6.25	0.062
	Age	1.01	0.97–1.04	0.673
	Sex	2.13	0.86–5.30	0.104
	TTO (vs. BSO)	0.95	0.36–2.50	0.909
	EVT ± IV thrombolysis	1.50	0.61–3.65	0.376
	PC-ASPECTS (per 1 point decrease)	0.93	0.65–1.33	0.695
	BATMAN (per 1 point decrease)	1.19	0.94–1.49	0.150
**Model 4**	Subset cranial and cerebellar ≥ 4	1.73	0.64–4.69	0.285
	Age	1.01	0.98–1.05	0.542
	Sex	0.44	0.18–1.07	0.069
	TTO (vs. BSO)	0.85	0.33–2.18	0.729
	EVT ± IV thrombolysis	1.57	0.63–3.94	0.333
	PC-ASPECTS (per 1 point decrease)	0.99	0.71–1.39	0.963
	BATMAN (per 1 point decrease)	1.15	0.92–1.45	0.229

OR, odds ratio; CI, confidence interval; NIHSS, National Institutes of Health Stroke Scale; TTO, truncal-type occlusion; BSO, branching-site occlusion; EVT ± IV thrombolysis, endovascular treatment with or without intravenous thrombolysis; PC-ASPECTS, Posterior Circulation-Alberta Stroke Program Early Computed Tomography Score; BATMAN, Basilar Artery on Computed Tomography Angiography score.

**Table 3:** Comparison of patients who experienced neurological deterioration and who did not, in those that did not undergo endovascular treatment.

Variables	ND (*N* = 13)	No-ND (*N* = 60)	*p* Value
**Age**	73 (48–77)	69.5 (56–77.75)	0.375
**Sex, male**	11 (84.6%)	43 (71.7%)	0.335
**HTN**	9 (69.2%)	35 (58.3%)	0.467
**DM**	7 (53.8%)	19 (31.7%)	0.130
**Atrial fibrillation**	1 (7.7%)	18 (30.0%)	0.097
**Presence of prodrome**	6 (46.2%)	7 (11.7%)	0.003
**Onset-to-door time (h)**	5 (2–28.5)]	7 (3–24)	0.870
**NIHSS at presentation**	7 (2.5–13.5)	4.5 (1.25–16.75)	0.438
**Subset mental status scores**	0 (0–2)	1 (0–3.75)	0.577
**Subset** **motor** **scores**	2 (0–5)	0 (0–4.75)	0.318
**Subset cranial and cerebellar scores**	3 (2–5)	2.5 (1–5.75)	0.934
**Occlusion degree**			0.020
Complete occlusion	7 (53.8%)	50 (83.3%)	
Incomplete occlusion	6 (46.2%)	10 (16.7%)	
**Occlusion location**			0.250
distal BA	0 (0.0%)	8 (13.3%)	
proximal BA	5 (38.5%)	27 (45.0%)	
VA	8 (61.5%)	25 (41.7%)	
**Occlusion type**			0.018
Truncal-type occlusion	13 (100.0%)	41 (68.3%)	
Branching-site occlusion	0 (0.0%)	19 (31.7%)	
**PC-ASPECTS**	8 (7–9.5)	9.5 (8–10)	0.144
**BATMAN**	5 (3.5–6)	6 (5–8)	0.077
**Final PC-ASPECTS**	8 (6–9.5)	9 (7–10)	0.935
**Good outcomes (mRS 0–2)**	2 (15.4%)	38 (63.3%)	0.002

Numbers are represented by numbers (percentage), median value [interquartile range]; HTN, hypertension; DM, diabetes mellitus; NIHSS, National Institutes of Health Stroke Scale; BA, basilar artery; VA, vertebral artery; PC-ASPECTS, Posterior Circulation-Alberta Stroke Program Early Computed Tomography Score; BATMAN, Basilar Artery on Computed Tomography Angiography score; mRS, modified Rankin scale.

**Table 4:** Logistic regression model for prediction of neurological deterioration in vertebrobasilar occlusion patients that did not undergo endovascular treatment.

Variables	OR	95% CI	*p* Value
Incomplete occlusion (vs. complete occlusion)	6.17	1.11–34.25	0.037
BATMAN (per 1 point decrease)	1.91	1.17–3.11	0.009
PC-ASPECTS (per 1 point decrease)	1.96	1.11–3.48	0.021
Age	0.96	0.91–1.02	0.208
Onset-to-door time (h)	0.97	0.94–1.00	0.087
NIHSS at presentation (per 1 point increase)	0.88	0.77–1.01	0.072

OR, odds ratio; CI, confidence interval; BATMAN, Basilar Artery on Computed Tomography Angiography score; PC-ASPECTS, Posterior Circulation-Alberta Stroke Program Early Computed Tomography Score; NIHSS, National Institutes of Health Stroke Scale.
